# Tumour–host divergence: a hypothesis for personalised blood-based early detection of hepatocellular carcinoma

**DOI:** 10.3389/fonc.2026.1932525

**Published:** 2026-09-17

**Authors:** Myoung-Eun Han, Hyo Jin Kim, Hansol Park, Sae-Ock Oh

**Affiliations:** 1 Department of Anatomy, School of Medicine, Pusan National University, Yangsan, Republic of Korea; 2 Research Center for Molecular Control for Cancer Cell Diversity, Pusan National University, Yangsan, Republic of Korea

**Keywords:** alpha-fetoprotein, anomaly detection, early detection, hepatocellular carcinoma, liquid biopsy, metabolic dysfunction-associated steatotic liver disease, surveillance, tumour microenvironment

## Abstract

Hepatocellular carcinoma (HCC) remains lethal because most tumours are found too late for cure, and the blood biomarkers used for surveillance — chiefly alpha-fetoprotein (AFP) — miss many early-stage and AFP-negative tumours; despite three decades of discovery, single-analyte and marker-plus-AFP panels have converged on a performance ceiling. We advance the tumour–host divergence (THD) hypothesis: that early malignant emergence is better detected as a personalised anomaly than as a population classification, and that pairing tumour-shed signals with the host’s immune–inflammatory response — read along each patient’s own trajectory and conditioned on aetiology — can reveal small tumours that any single marker, measured once against a population threshold, will miss. The hypothesis rests on three complementary axes: a temporal axis interpreting change from the individual’s baseline rather than a fixed cut-off; a compartmental axis treating the coupling between tumour-shed and host-response signals as the quantity of interest, on the premise that a small tumour may perturb the host before it sheds detectable marker; and a contextual axis conditioning interpretation on aetiology and fibrosis stage, including metabolic dysfunction-associated steatotic liver disease. These axes are integrated by a per-patient longitudinal risk model that flags malignant emergence as a departure from the patient’s stable equilibrium. To show that this gap is real rather than assumed, we map the field across a de-duplicated corpus of 8,069 PubMed and Embase records with an explicit, reproducible taxonomy, finding research effort concentrated on tumour-derived analytes — which outnumber host-response analytes by roughly six and a half to one — far more work on prognosis than early detection, and the temporal, paired-compartment and aetiology-conditioned dimensions almost unexplored. The hypothesis is deliberately falsifiable: its central predictions are benchmarked against GALAD and are testable in surveillance cohorts that already generate longitudinal data. Its contribution is integrative — reorganising existing biomarkers rather than requiring new ones — and is aimed at the early-stage and AFP-negative patients of greatest unmet need.

## 1 Introduction

HCC is among the leading causes of cancer death worldwide, and outcome is dominated by stage at diagnosis: small, solitary tumours are amenable to resection, ablation or transplantation, whereas advanced disease is treated palliatively. Effective surveillance of at-risk populations — predominantly patients with cirrhosis or chronic viral hepatitis — therefore offers the largest achievable reduction in mortality (7, 36). In practice, surveillance rests on semi-annual ultrasonography with serum AFP, a combination whose sensitivity for early-stage disease is modest. AFP is normal in a substantial proportion of HCC, and the so-called AFP-negative tumours — roughly a third of cases — are precisely those for which a complementary blood test is most needed (2–4). The clinical problem is therefore not a shortage of candidate markers but a shortage of markers that detect tumours early and that perform in the patients AFP misses.

To take stock, we assembled the published literature indexed under HCC blood diagnostic biomarkers from two databases and de-duplicated it before analysis. The PubMed export contained 6,711 records and the Embase export 2,236, a pooled total of 8,947. After removing 878 duplicate records — two redundant entries within PubMed and 876 Embase records already represented in PubMed or internally — 8,069 unique records remained. We classified each record by analyte class and clinical framing using a transparent title- and abstract-keyword taxonomy of twenty-three biomarker groups (**Figure 1A**; the de-duplication workflow is summarised in **Supplementary Figure 1**). Because this taxonomy is deliberately more granular than the broad groupings used in some earlier surveys, and because it operates on the de-duplicated rather than the raw corpus, the absolute counts differ from naïve database tallies; the qualitative imbalances, however, are robust. To keep the classification auditable, the exact search strings, database export dates and the full twenty-three-class keyword dictionary are provided in the **Supplementary Material**, and its reliability against full-text reading is quantified there (**Supplementary Table 1**). One direction of error is worth stating at the outset: because host-response work is frequently described by disease- or assay-specific terms that do not surface in a title or abstract (for example a complete-blood-count ratio reported within a prognostic study), a keyword taxonomy is more likely to under-count than to over-count the host-response compartment. The tumour-versus-host imbalance reported here is therefore, if anything, a conservative estimate of the true skew.

**Figure 1:**
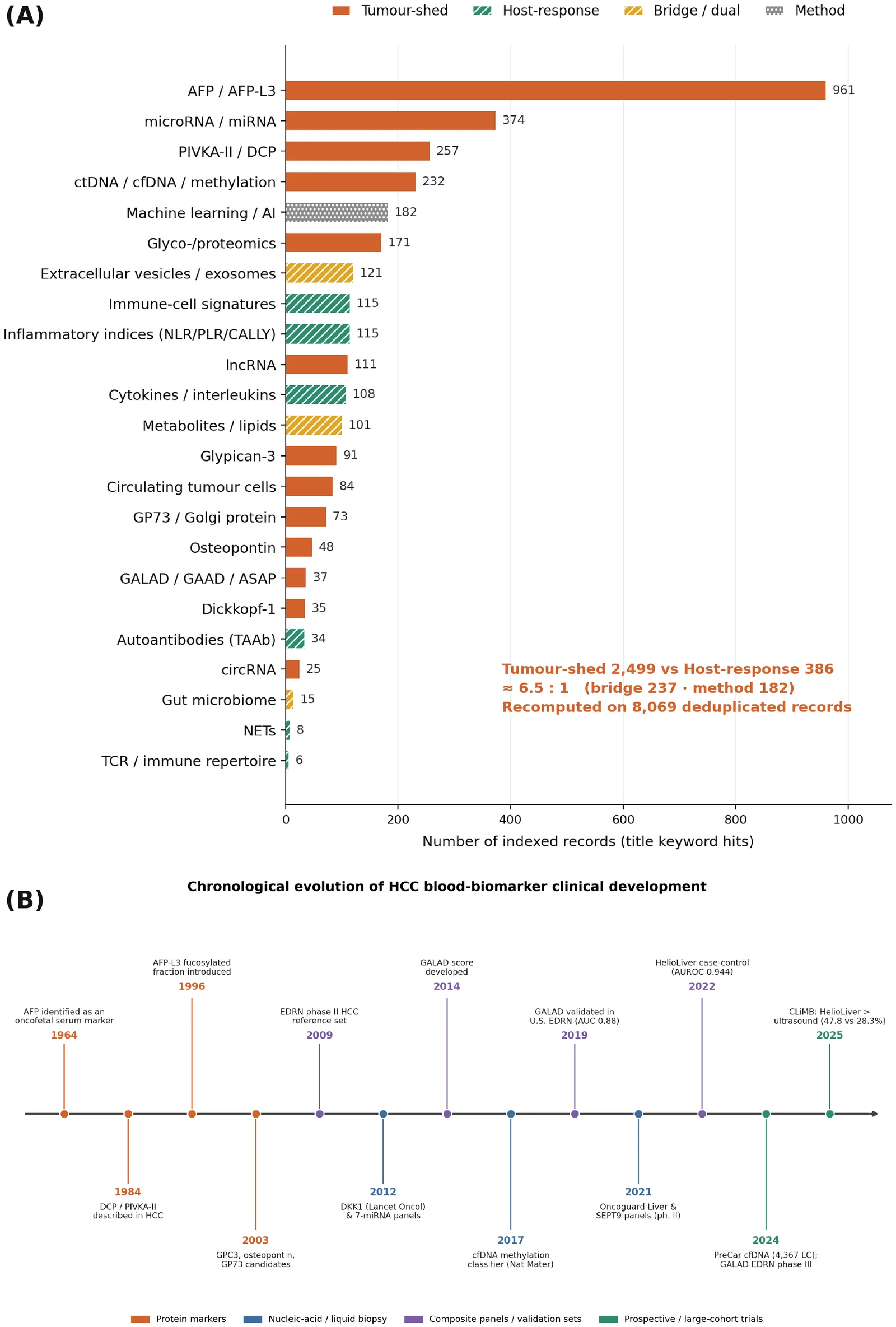
**(A)** Research volume of candidate HCC blood biomarkers by class. Number of indexed records (title- and abstract-keyword hits across the 8,069 de-duplicated PubMed and Embase entries) for each biomarker class, ranked in descending order and coloured by tumour–host divergence compartment: tumour-shed (solid), host-response (forward hatch), bridge/dual-compartment (forward hatch, gold) and methodological (dotted). AFP-centred markers dominate (n = 961; ≈2.6× the next class), and tumour-shed markers collectively outnumber host-response markers by roughly six and a half to one (2,499 vs 386; bridge 237, method 182). The imbalance describes how research attention is distributed rather than how strong the biological evidence is, and quantifies the under-exploration of the host-response axis and of paired tumour×host measurement. Counts were recomputed on the de-duplicated corpus using a 23-class taxonomy and therefore differ from raw database tallies; the qualitative pattern is unchanged. **(B)** Chronological development of HCC blood biomarkers, 1964–2025. Milestones are coloured by type — single protein marker, nucleic-acid/circulating-DNA marker, composite or multi-analyte panel, and pivotal prospective study — and span the discovery of AFP through the contemporary multi-analyte and prospective-validation era. Markers accumulate in waves, each adding a biological layer rather than displacing the previous one.

Two imbalances stand out. First, prognosis is the dominant theme, appearing roughly 2.6-fold more often than early detection — even though early detection is where survival is decided. Second, the marker landscape is overwhelmingly tumour-centric: AFP-related work alone accounts for 961 records, approximately 2.6 times the next most studied class, and tumour-derived analytes collectively outnumber host-response analytes by approximately six and a half to one (2,499 versus 386; **Figure 1A**). Within this large literature, the AFP-negative subgroup, the metabolic (MA SLD) aetiology, and — most strikingly — any longitudinal, within-patient or paired tumour–host analysis are each represented in well under 1% of titles. These counts indicate where research effort has been concentrated; they do not, by themselves, measure the strength of the underlying biological evidence. A frequently studied marker class may consist of many small reports, whereas another may be supported by fewer, larger studies. The counts therefore identify an imbalance in investigative attention. Because the keyword approach is also likely to miss some host-response studies, the limited attention to host biology and paired-compartment measurements is probably not overstated.

In this review we (i) summarise the current blood-biomarker landscape, organised not by molecule but by the biological compartment each marker reports on, with each major class examined for its advantages, disadvantages and limitations; (ii) trace the chronological development of the field and its pivotal clinical studies; (iii) compare HCC biomarkers with the better-established blood biomarkers of hepatitis and cirrhosis from which they must be distinguished; (iv) argue that the performance plateau is the predictable consequence of three shared structural assumptions; and (v) propose the tumour–host divergence framework, which reconceives blood biomarkers along temporal, compartmental and contextual axes and reframes early detection as personalised anomaly detection. We close with testable predictions, study designs and the principal barriers to implementation.

## 2 The current landscape of blood biomarkers

Across the de-duplicated corpus, candidate markers are distributed very unevenly among classes (**Figure 1A**). To make this landscape interpretable we group circulating markers not by molecule but by the biological compartment each interrogates (**Figure 2**; **Table 1**): tumour-shed signals released by malignant hepatocytes, host-response signals generated by the immune–inflammatory reaction to the tumour, and a small set of bridge analytes that carry information from both compartments. A fourth, methodological category captures the machine-learning and multi-omics approaches used to combine markers. The sections below treat each compartment in turn; within the tumour-shed compartment, which dominates the literature, we subdivide further by molecular class and tabulate the advantages, disadvantages and limitations of each group.

**Figure 2:**
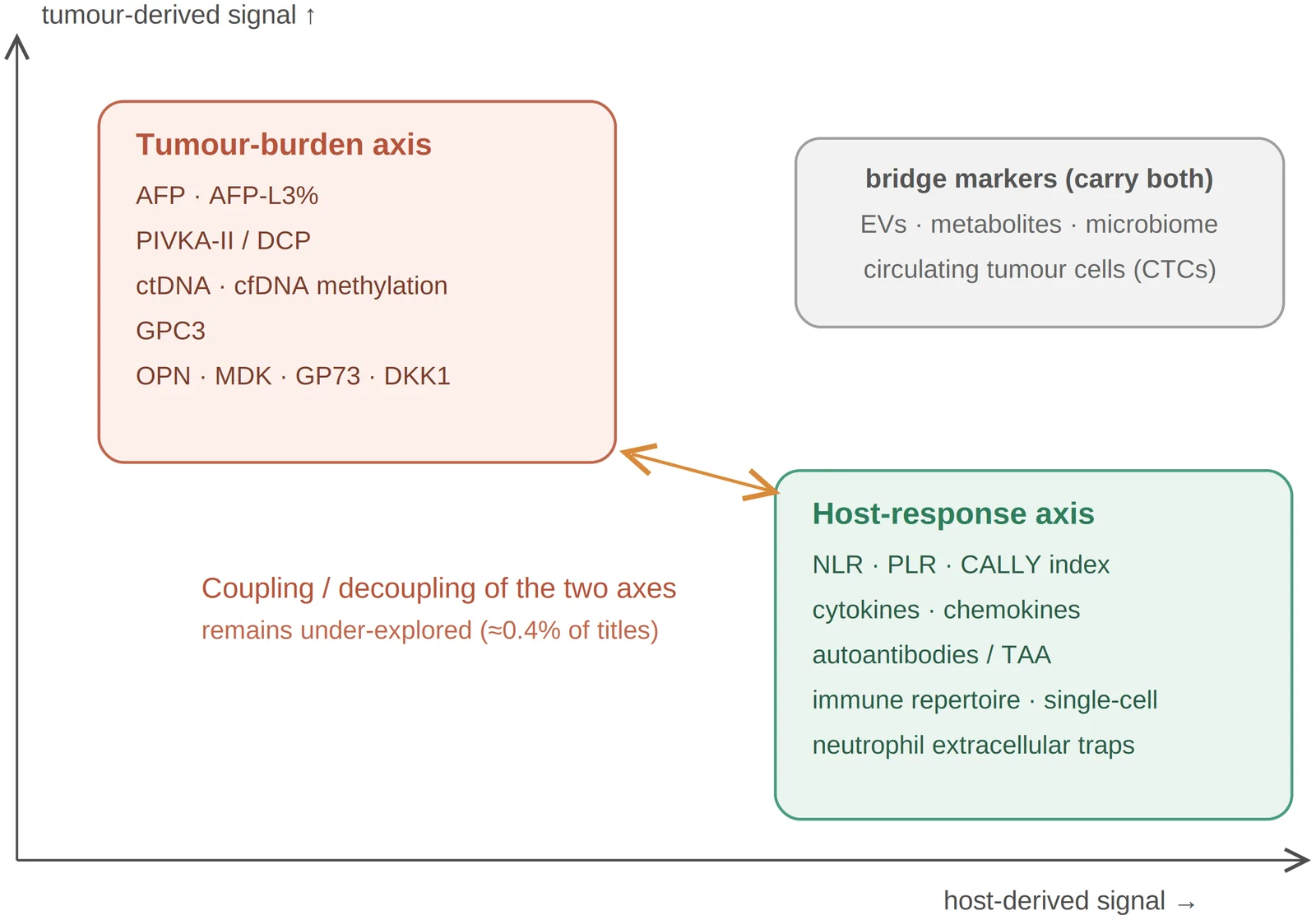
Re-classification of existing HCC blood biomarkers along tumour-derived and host-derived axes. Tumour-burden markers (upper-left) are shed or released by malignant hepatocytes; host-response markers (lower-right) report the systemic immune–inflammatory reaction; bridge analytes (centre) carry information from both. The diagonal coupling/decoupling of the two axes — their joint, paired measurement — is the principal under-explored opportunity. Circulating tumour cells are placed with tumour-burden markers in the figure for consistency with common classification, although their interactions with host cells mean that they can also be viewed as bridge analytes (Section 2.1).

**Table 1:** The blood-biomarker landscape in HCC, organised by compartment and analyte class.

Marker/signal	Biological basis	Principal strength/limitation
Tumour-shed — oncofetal and coagulation proteins, and composite scores		
AFP	Oncofetal glycoprotein re-expressed by a subset of HCC	Normal in ~⅓ of HCC; non-specific in hepatitis/cirrhosis
AFP-L3	Tumour-specific fucosylated AFP fraction	Unreliable when total AFP is low
PIVKA-II/DCP	Under-carboxylated prothrombin from malignant hepatocytes	Confounded by vitamin K status, warfarin, cholestasis
GALAD/GAAD/A SAP	Logistic combination of the above with age and sex	Population-thresholded; aetiology-blind; phase-3 early sensitivity ~62–65%
Tumour-shed — other secreted and tumour-associated proteins		
Glypican-3	Heparan-sulfate proteoglycan re-expressed in HCC	Specific vs benign nodules; weak in small tumours
Osteopontin	Adhesion/invasion phosphoprotein	Complements AFP; rises in benign liver disease
Dickkopf-1	Wnt-pathway antagonist	Value in AFP-negative disease; variable cut-offs
GP73 (GOLM1)	Golgi membrane protein	Sensitive; poor specificity in chronic hepatitis
Midkine/ SCCA	Growth factor/serpin	Signal in AFP-negative HCC; limited validation
Tumour-shed — circulating tumour DNA		
Somatic mutations	TERT, TP53, CTNNB1 variants	Highly specific; sparse in early disease
Methylation	Locus-specific 5-methylcytosine	Abundant, tissue-informative; background drift
5-hydroxymethylcytosine	Genome-wide 5hmC regulatory marks	Strong early signal; specialised assay
Fragmentomics	Fragment length, motifs, nucleosome footprint	Depth-robust, complementary; complex bioinformatics
Tumour-shed — circulating non-coding RNA		
microRNA	7-miRNA serum panels	Stable, AFP-negative value; normalisation variance
lncRNA	Diagnostic lncRNA networks	Tissue-of-origin signal; poor cross-study overlap
circRNA	Exonuclease-resistant closed loops	High stability; early-stage evidence immature
Host-response markers		
Inflammatory indices	NLR, PLR, SII, CALLY	Free, universal; non-specific
Cytokines	IL-6, IL-10, VEGF, TGF-β	Mechanistic, adds to AFP; short half-life
Autoantibodies	Anti-BIRC5/TAA panels	Stable, AFP-negative value; low single-marker sensitivity
Immune repertoire	TCR/BCR sequencing	Reads adaptive response; large between-person variation
Cellular signatures	MD SC, Treg, NK, single-cell	Functional, predictive; costly, low-throughput
Macrophages (TAM)	Dominant TME immune population; M2 polarisation	Central to immune escape and immunotherapy response; assayed mainly in tissue
NETs	cfDNA–MPO, citH3	Novel mechanism; unproven diagnostically

This table consolidates the marker groups discussed in Section 2 (previously presented as separate tables for each class). Strengths and limitations are summarised qualitatively; per-marker performance varies with assay, cohort and disease stage.

### 2.1 Tumour-shed markers

Markers in this compartment derive directly from the tumour and account for the great majority of the field. The oncofetal and coagulation-related proteins — AFP, its fucosylated fraction AFP-L3, and PIVKA-II/DCP — remain the clinical mainstays, and their logistic combination with age and sex (GALAD, and the related GAAD and A SAP scores) is the current benchmark, although all are population-thresholded and aetiology-blind (5, 6). A second tier of secreted and tumour-associated proteins (glypican-3, osteopontin, Dickkopf-1, GP73, midkine, SCCA) adds incremental and often AFP-negative signal but is limited by specificity in chronic liver disease and uneven validation (2–4, 8–11). Circulating tumour DNA contributes complementary genetic and epigenetic signals — somatic mutations, locus-specific methylation, genome-wide 5-hydroxymethylcytosine and fragmentomic features — that are highly specific but sparse in early disease (1, 33). Circulating non-coding RNAs (microRNA panels, diagnostic lncRNA networks and exonuclease-resistant circRNAs) are stable and retain value when AFP is normal, but suffer normalisation variance and limited cross-study reproducibility (12–16, 32). Circulating tumour cells provide whole-cell material for morphology and culture. Although uncommon in early HCC (29), they also provide information that is not confined to the tumour compartment: circulating tumour cells undergo epithelial–mesenchymal plasticity and interact with circulating immune cells. Their phenotype can therefore reflect both tumour and host biology, making them reasonable candidates for the bridge category rather than a strictly tumour-shed class (47). Glycoproteomic and proteomic signatures offer high-dimensional discrimination at the cost of assay complexity (25–27).

### 2.2 Host-response markers

A smaller but mechanistically distinct compartment reports on the host reaction to the tumour rather than the tumour itself. Composite inflammatory indices from routine blood counts (NLR, PLR, SII, CALLY) are free and universal but non-specific (17, 18); some of these indices also have predictive value for immunotherapy response in HCC, so the same host-response measurements may contribute to the stratification component of the framework (Section 5.5); circulating cytokines and growth factors (IL-6, IL-10, VEGF, TGF-β) are mechanistically informative and complement AFP but have short half-lives (19); and tumour-associated autoantibodies are stable and retain value in AFP-negative disease despite low single-marker sensitivity (3). Deeper adaptive-immune readouts — T-cell and B-cell receptor repertoire sequencing (20), cellular immune signatures (tumour-associated macrophages, MD SCs, regulatory T cells, NK cells, single-cell profiles) (21), and neutrophil extracellular traps (22) — capture the host response at increasing biological resolution, but with large between-person variation and, as yet, limited diagnostic validation. Tumour-associated macrophages are especially relevant in this setting. They constitute a major immune component of the HCC microenvironment and participate in immune escape and checkpoint-therapy response. Most measurements are tissue based, but circulating or secreted correlates of macrophage activity could provide related information on the host-response side (44, 45).

### 2.3 Bridge analytes and integrative methods

Some analytes report on both compartments at once: extracellular vesicles and exosomes carry tumour-derived cargo within a host-regulated vehicle (12, 13); circulating metabolites and lipids reflect tumour metabolism read out through host physiology (23, 24); and gut-microbial signatures index a systemic host–environment axis (30). Integrative computational methods — multi-marker machine-learning and multi-omics models — combine these inputs and consistently outperform single analytes, though at the cost of interpretability and prospective validation (18, 27, 28).

### 2.4 Synthesis of the landscape

Read across the three compartments, the de-duplicated corpus tells a consistent story. The tumour-shed compartment (2,499 records) contains every clinically established marker and almost all recent innovation, yet is bounded by a single biological ceiling — signal scales with tumour burden, so the compartment is weakest in exactly the small, early, AFP-negative tumours that define the unmet clinical need. The host-response compartment (386 records) is studied roughly six-and-a-half times less, despite possessing the one property the tumour compartment lacks: the capacity to amplify a small malignant stimulus and therefore to register change before appreciable shedding occurs. The bridge compartment (237 records) and methodological category (182 records) offer native routes to combine information, but are presently applied in the same cross-sectional, population-referenced manner as everything else.

Three observations recur at every level of this taxonomy and are not artefacts of any single class. First, almost every marker is studied alone, against a population threshold, at a single time point. Second, the markers that are biologically best positioned for early detection — host-response signals and paired tumour×host measurements — are the least studied. Third, the headline performance of the most promising tumour-shed platforms is reported overwhelmingly in case-control designs, which systematically overstate prospective utility (Section 2). These three observations are the empirical basis for the structural argument that follows: the plateau is a property of measurement convention, not of the molecules themselves.

It is worth stating plainly what this synthesis does and does not claim. It does not claim that any individual marker is poor; many are excellent within their validated context. It claims that the field’s collective design choices — static, single-compartment and context-blind measurement — have been applied so uniformly that adding further markers within those choices yields diminishing returns. The remedy, developed in Section 5, is not a new molecule but a new way of using the molecules already in hand.

### 2.5 Chronological development and pivotal studies

HCC blood biomarkers have evolved in identifiable waves, each adding a layer of biological information rather than replacing the last: from single oncofetal proteins (AFP from the 1960s, then PIVKA-II and AFP-L3), through secreted-protein candidates, to composite serological algorithms, and most recently to multi-analyte tests that fuse circulating-DNA features with proteins. **Figure 1B** places the principal milestones on a timeline and **Table 2** summarises the pivotal studies; to avoid duplication we highlight only the interpretive thread here. Performance is best read through the Early Detection Research Network (EDRN) five-phase framework (37), which separates case-control evaluation (phase 2) from retrospective longitudinal (phase 3) and prospective (phase 4) validation, because the same marker typically looks stronger in phase 2 than in prospective use. The composite GALAD score, developed in 2014 (38) and later validated in the United States with the ultrasound-augmented GALADU S configuration (39), illustrates this: a prospective EDRN phase 3 study reported sensitivity of roughly 62% within 12 months of diagnosis at 82% specificity — more sober than case-control estimates but superior to AFP (40). The multi-analyte era extended this trajectory, with methylation-plus-protein classifiers such as HelioLiver reporting case-control AUROCs near 0.94 (41) and commercial methylated-DNA assays (Oncoguard Liver (42); methylated SEPT9 (43)) entering phase-2 evaluation.

**Table 2:** Pivotal milestones in HCC blood-biomarker development.

Year	Milestone	Significance/evidence level
1964	AFP linked to hepatoma	First serum HCC marker; foundation of surveillance
1984	PIVKA-II/DCP described	Biologically independent coagulation-related marker
1996	AFP-L3 fraction	Tumour-specific fucosylated glycoform; greater specificity
~2003	GPC3, OPN, GP73	Wave of secreted-protein candidates (mostly phase 1–2)
2009	EDRN reference specimen set	Enabled standardised phase-2 case-control comparison
2012	Dickkopf-1; multi-miRNA panels	AFP-negative value; non-coding RNA era begins
~2014	GALAD developed	Reference composite serological algorithm
2017	cfDNA methylation classifier	Opens circulating-DNA multi-analyte era
2019	GALAD U S validation	AUC ~0.88; phase-2/3 evidence
2021	Methylation + AFP commercial tests	e.g. ~88% sens at ~87% spec (phase 2)
2022	Methylation+protein classifier	Case-control AUROC ~0.94 > GALAD ~0.90 > AFP ~0.85
2024	GALAD phase-3; large cfDNA-feature cohorts	Prospective sens ~62–65%; cfDNA early-stage sens ~50%
2025	Prospective multi-analyte screening trial	Higher early-stage sensitivity than ultrasound (phase 4)

Performance figures are approximate and depend strongly on study design; case-control (phase 2) estimates typically exceed prospective (phase 3–4) estimates for the same marker.

## 3 Why performance has plateaued

Three assumptions are shared across most of the literature and, we argue, jointly explain the plateau. They are not properties of any particular molecule but of how molecules are measured and interpreted, which is why adding new markers within the same framework has yielded diminishing returns. Each assumption is examined below with concrete examples, and we give particular attention to where each fails most severely: metabolic dysfunction-associated steatotic liver disease (MA SLD).

### 3.1 The static assumption: a single measurement against a fixed threshold

In the prevailing paradigm a marker is drawn once and compared with a fixed population threshold, discarding the most informative reference available in a surveilled population — the patient’s own previous values. HCC arises over years against a chronic-liver-disease background, yet cross-sectional testing ignores individual trajectory (36). The cost of this is concrete. Consider a patient whose true personal AFP baseline is 3 ng/mL and who, as a tumour emerges, rises to 18 ng/mL: this six-fold personal increase is biologically alarming yet sits comfortably below the conventional 20 ng/mL cut-off and is declared normal. Conversely, a patient with active hepatitis whose stable baseline is 30 ng/mL is repeatedly flagged as abnormal despite no change. Because within-person variation is far smaller than between-person variation, the fixed threshold simultaneously misses true emergences and generates false alarms. The widespread availability of serial surveillance data makes this discarded information especially regrettable.

### 3.2 The single-compartment assumption: tumour or host, never both

Almost every study reports either a tumour-shed or a host-response signal, not both, and so cannot capture the asymmetry that defines early disease — a small tumour that sheds little but has already begun to perturb the host. The corpus makes this stark: tumour-shed and host-response markers are studied largely in separate literatures, and their joint, simultaneous measurement appears in well under 1% of titles. Yet the biology is sequential: a 1 cm tumour may release too little AFP or ctDNA to cross any threshold while already provoking measurable shifts in inflammatory indices, cytokines or autoantibody titres. By measuring only one axis, conventional designs are blind to precisely the decoupling that offers the earliest window. This is not a limitation of the markers — both axes already exist — but of the convention of studying them apart.

### 3.3 The context-blind assumption: one cut-off for every aetiology and fibrosis stage

A single population cut-off is applied regardless of aetiology and fibrosis stage, even though the baseline distributions of AFP, inflammatory indices and cytokines differ markedly between hepatitis B, hepatitis C, alcohol-related and metabolic liver disease. AFP runs higher on average in active hepatitis B and during hepatitis flares; it is lower and less informative in many MA SLD patients; inflammatory indices shift with the degree of portal hypertension and fibrosis. A threshold calibrated on a mixed population is therefore mis-calibrated for every individual subgroup, over-calling in high-baseline aetiologies and under-calling in low-baseline ones.

This assumption fails most severely in MA SLD-associated HCC, which is the fastest-growing aetiology, is frequently non-cirrhotic, and tends to be AFP-poor (18, 31). Patients with metabolic liver disease often fall outside cirrhosis-based surveillance criteria, present with tumours that shed little AFP, and carry metabolic comorbidities that further distort population-referenced markers. A framework that conditions interpretation on aetiology and fibrosis stage — and that treats MA SLD as a distinct context rather than an afterthought — is therefore not a refinement but a necessity. Sections 4 and 5 develop the aetiology-conditioned view, first by comparing HCC markers with the fibrosis and viral markers that define these contexts, then by formalising context as an explicit axis of the proposed framework.

## 4 Comparison with blood biomarkers of hepatitis and cirrhosis

HCC almost always arises within a diseased liver, so its blood biomarkers operate against a background defined by the markers of viral hepatitis and of fibrosis/cirrhosis. Understanding that background is essential for two reasons: these markers define the surveillance population and the aetiologic context that Section 3 showed to be decisive, and they provide an instructive contrast in maturity. The fibrosis markers are, in several respects, better validated and more guideline-embedded than most HCC early-detection markers — a gap worth understanding (**Table 3**).

**Table 3:** Blood biomarkers of viral hepatitis, fibrosis/cirrhosis and HCC compared.

Domain	Representative markers	Performance/role	Maturity vs HCC markers
Viral hepatitis	HBsAg, HBV-DNA, HCV-RNA	Diagnose infection, quantify activity, set risk	Mature; define surveillance entry
Simple fibrosis indices	FIB-4, APRI	AUROC ~0.8–0.85 for advanced fibrosis	Mature, free, widely used
Advanced fibrosis panels	ELF, FibroTest, NF S	ELF AUROC ~0.92 for cirrhosis	Guideline-recognised
Bridging risk scores	aMAP ( ± longitudinal ΔaMAP)	HCC risk + cirrhosis (AUROC ~0.79)	Spans fibrosis→cancer risk
HCC tumour markers	AFP, GALAD, multi-analyte	Prospective early sens ~50–65%	Less mature prospectively

The fibrosis and viral markers define the aetiology-conditioned context within which HCC markers must be interpreted, and several are more mature than current HCC early-detection markers (**Table 3**).

### 4.1 Viral hepatitis markers define the at-risk population

Chronic hepatitis B and C are leading causes of HCC, and their blood markers — hepatitis B surface antigen (HBsAg) and HBV-DNA, and HCV antibody and HCV-RNA — establish aetiology and viral activity. They are diagnostic of infection and quantify replication, and HBV-DNA level and HCV cure status modify HCC risk: sustained virological response in hepatitis C lowers but does not eliminate risk, leaving a population that still requires surveillance. These markers are not HCC markers, but they are the entry criterion for surveillance and a primary determinant of the aetiology-conditioned baseline ( Section 3.3).

### 4.2 Fibrosis and cirrhosis markers grade the substrate

The degree of fibrosis is the strongest structural determinant of HCC risk, and blood-based fibrosis markers are comparatively mature. Simple indices combine routine tests: FIB-4 (age, A ST, ALT, platelets) and APRI (A ST-to-platelet ratio) are inexpensive screens with AUROCs around 0.8–0.85 for advanced fibrosis or cirrhosis. Proprietary panels add accuracy: the Enhanced Liver Fibrosis (ELF) test, which measures three direct matrix-turnover analytes, reaches AUROCs around 0.92 for cirrhosis — exceeding FIB-4 — and is recognised in several guidelines; FibroTest and the NAFLD fibrosis score occupy similar niches. These markers report the host’s fibrogenic response rather than any tumour, but they grade exactly the substrate on which HCC develops.

### 4.3 Bridging scores: from fibrosis grading to HCC risk

A particularly instructive class bridges fibrosis assessment and HCC risk prediction. The aMAP score combines age, sex, albumin–bilirubin and platelets to estimate HCC risk across aetiologies; notably, the same inputs also discriminate cirrhosis (AUROC ~0.79, exceeding FIB-4 and APRI), so a single score spans the fibrosis-to-cancer-risk continuum. The concept of a longitudinal change in such a score (for example ΔaMAP) is a direct analogue, in the fibrosis domain, of the personal-trajectory thinking this review advocates for tumour markers — evidence that the temporal, individualised framing is already gaining traction in adjacent fields.

Two lessons follow from the comparison. First, the fibrosis markers show that composite, routinely available blood panels can achieve guideline-grade performance — a standard HCC early-detection markers have largely not yet met in prospective use. Second, because fibrosis markers report the host substrate while HCC markers (mostly) report the tumour, the two are naturally complementary: the fibrosis/host axis sets the context and risk, and the tumour axis signals emergence. This complementarity prefigures the compartmental and contextual axes of the framework developed next.

## 5 The tumour–host divergence (THD) framework

### 5.1 From classification to anomaly detection

The prevailing paradigm asks a classification question: does this measurement resemble the “cancer” or the “no-cancer” class? For early tumours the answer is intrinsically uncertain, because their values lie near the decision boundary where the benign and malignant population distributions overlap. We propose instead an anomaly-detection question: has this individual departed from their own stable equilibrium? In a surveillance programme, repeated measurements make each patient their own control; because within-person variation is far smaller than between-person variation, a deviation invisible against the population spread can be conspicuous against the patient’s baseline. **Figure 3A** renders this as a two-dimensional phase plane whose axes are the tumour-shed and host-response signals: a tumour-free patient occupies a stable region, and malignant emergence is, in this model, a characteristic divergence that the framework predicts may register on the host-response axis before it crosses the conventional tumour-marker threshold.

**Figure 3:**
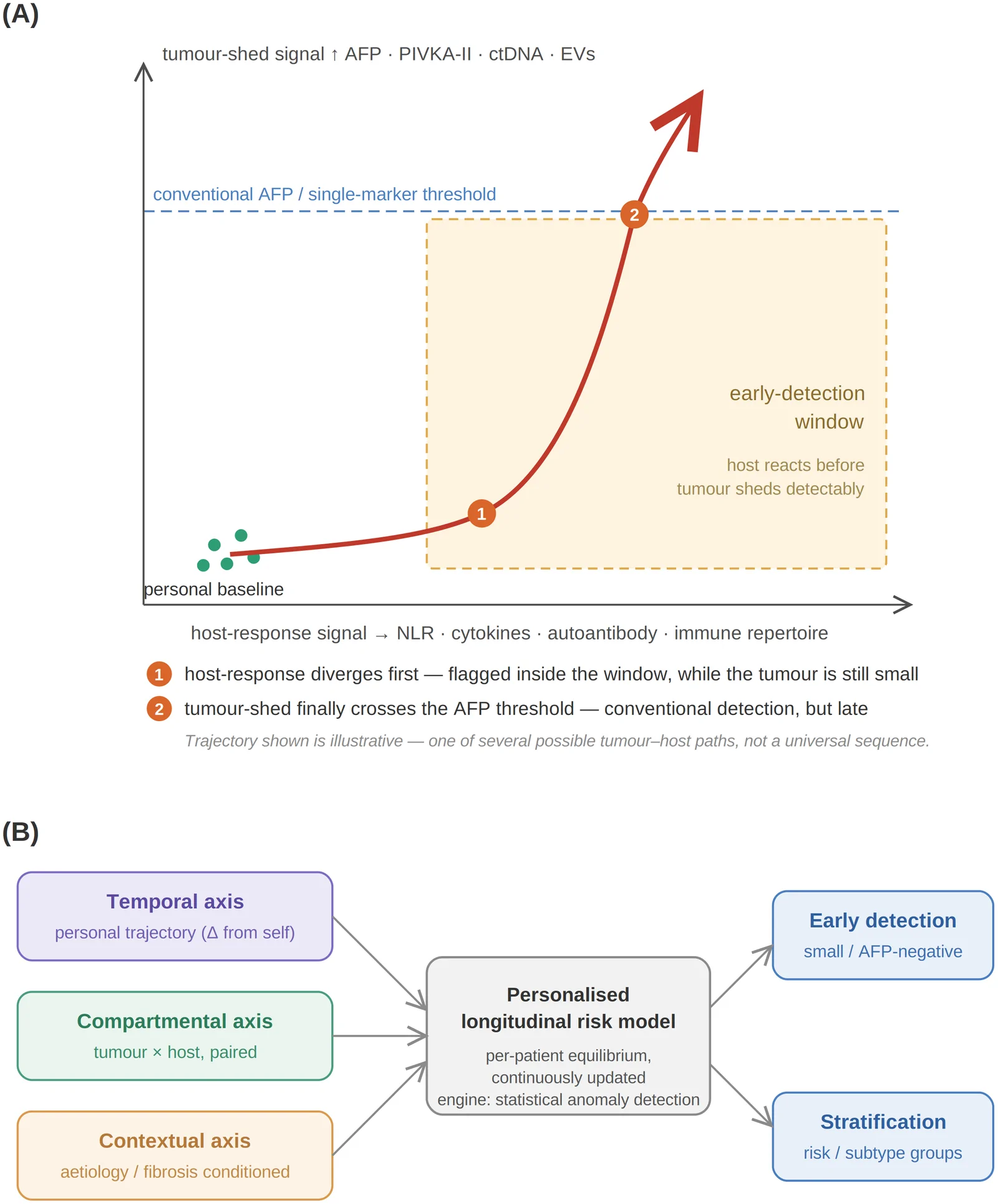
The tumour–host divergence framework. **(A)** Tumour-shed and host-response measurements define a two-dimensional state space. During a tumour-free period, repeated observations define a stable personal region. Malignant emergence is hypothesised to move the patient away from this region; in some patients, the host-response component may change before a conventional tumour-marker threshold is crossed. The illustrated path is only one possibility: host-first, tumour-first, synchronous and weak-response patterns are all compatible with the framework. **(B)** The temporal, compartmental and contextual information is combined in a per-patient statistical model. Each new measurement is evaluated against an aetiology-conditioned personal reference, with the intended outputs of earlier detection and improved stratification. The central node represents a continuously updated statistical reference model, not a mechanistic simulation of hepatic physiology ( Section 5.5).

It is important to be precise about what in this proposal is new and what is not, because each ingredient has a literature of its own. Longitudinal biomarker monitoring, personalised baselines, anomaly detection and multi-omics integration are all established ideas, and adaptive risk models such as longitudinal applications of GALAD and the change in aMAP (ΔaMAP) already update an individual’s risk over time. We do not claim to have invented these primitives. The framework’s contribution is the particular way it combines them, and in particular one element that existing longitudinal models largely omit. Those models track a single compartment — almost always a tumour-shed marker or a composite of tumour-shed markers — along the time axis; the tumour–host divergence framework instead treats the relationship between two biologically opposing compartments as the quantity of interest and reads their decoupling, conditioned on aetiology and fibrosis stage, as the early signal. The novelty is therefore not longitudinal monitoring as such, but monitoring the coupling between a tumour-burden axis and a host-response axis within an aetiology-specific personal baseline. Framed this way the proposal is an organising synthesis that carries an empirical commitment: if a paired, coupling-aware model adds nothing over a well-constructed single-compartment longitudinal model, the framework is wrong in its central claim, and future prospective studies are designed to detect exactly that.

### 5.2 Pillar 1 — the temporal axis (personal trajectory)

The first axis replaces the fixed cut-off with the patient’s longitudinal trajectory. Rather than asking whether AFP exceeds a universal value, it asks whether AFP and other markers have diverged from the individual’s established baseline — a change-point or velocity signal. The feasibility of this approach is supported by recent validation of longitudinal screening algorithms in cirrhosis (36) and by prospective designs that incorporate serial sampling (7); it also parallels the longitudinal fibrosis-score thinking noted in Section 4.3. This axis is almost absent from the existing literature and is, in our analysis, the single largest untapped opportunity.

### 5.3 Pillar 2 — the compartmental axis (paired tumour × host)

The second axis pairs the two compartments and interrogates their coupling. Because host-response signals may, for at least some tumours, rise before tumour-shed signals become detectable, the divergence — or decoupling — between the axes could itself be an early signal and might flag tumours too small to cross any single-marker threshold. We are explicit that the consistency and direction of this precedence are not established and that the supporting evidence is at present limited and largely indirect ( Section 6); the claim is biologically plausible and is advanced here as a hypothesis, not as a demonstrated fact. This sequence should not be assumed for every HCC. Depending on aetiology, immune phenotype, tumour size, fibrosis and the marker being measured, the trajectory may be host-first, tumour-first, synchronous or weak-response. The THD hypothesis therefore allows several tumour–host trajectories. Whether such patterns form reproducible dynamical phenotypes is a question for empirical testing. The components already exist on both axes ( Section 2); what is missing is their joint, simultaneous measurement, which appears in well under 1% of the literature. Pairing, for example, a tumour-shed panel with an autoantibody or immune-repertoire read-out (3, 20, 21) is therefore a low-cost, high-yield direction that requires recombination of existing assays rather than new discovery. This also gives a mechanistic answer to an obvious objection: why should adding host markers improve on an already-optimised composite such as GALAD? GALAD’s five inputs — AFP, AFP-L3, DCP, age and sex — are all oncofetal, coagulation-related or demographic correlates of tumour-shed biology, and they therefore share a common failure mode: each scales with tumour burden and each is weak in the small tumour. Inputs that share a failure mode are correlated, so combining more of them yields diminishing returns, which is consistent with the early-stage performance ceiling GALAD reaches in prospective use. A similar limitation is seen when additional tumour-derived variables are added to GALAD. TAGALAD and GAP-TALAD improve overall discrimination, but their performance in early-stage disease remains limited (46). This is compatible with diminishing gains when several inputs remain closely tied to tumour burden, and it defines the gap that the temporal and host-response components are intended to address. To improve discrimination, an added feature must contribute information that remains useful after tumour-burden markers, aetiology, fibrosis stage and clinical covariates have been considered. This does not require host-response and tumour-derived variables to be statistically independent. Inflammation, fibrosis, immune activation, metabolic dysfunction and tumour burden are biologically linked, and some correlation is expected. Even so, two sources may provide additional conditional information: the host-response compartment, which can react to a stimulus out of proportion to its size, and the temporal axis, which references a patient against their own past rather than against the population. The prediction that these inputs improve on GALAD is thus not the weak claim that “more markers are better” but the more specific and testable proposition that host-response and temporal measurements retain predictive information after the existing predictors have been taken into account; were the added markers to behave merely as further surrogates of tumour burden, the framework predicts no gain. Crucially, pairing the compartments also addresses the principal objection to host-response markers — their non-specificity. A cold, a cholangitis episode, a hepatitis flare or a change in alcohol intake raises host-inflammatory signals, and read alone these would generate false alarms. The framework discriminates such intercurrent events from incipient malignancy on two grounds it does not share with single-marker testing. First, coupling: an intercurrent inflammatory insult perturbs the host axis while leaving the tumour-shed axis at baseline, whereas emerging malignancy is predicted to produce a sustained alteration in the joint tumour–host trajectory that departs from the individual’s usual coupling pattern, so it is the joint trajectory rather than either marker that carries the signal. Second, temporality (Pillar 1): a self-limiting infection produces a transient host-axis excursion that returns to the personal baseline, whereas a tumour produces a sustained, progressive divergence. In this framework, then, specificity is intended to arise from the coupled, longitudinal pattern rather than from any individual analyte; the non-specificity of host markers in isolation is what the paired, trajectory-based reading is designed to overcome. We present this as a design hypothesis, not an established result. Whether coupling and temporality actually hold specificity at a clinically acceptable level against the frequent intercurrent events of cirrhosis — infection, alcohol, hepatitis flare, cholangitis, fluctuating metabolic disease — is itself one of the questions future prospective studies are designed to test, and Section 6 sets out the conditions under which these safeguards could fail.

### 5.4 Pillar 3 — the contextual axis (aetiology- and fibrosis-conditioned)

The third axis conditions all interpretation on the underlying liver disease. The “normal” manifold differs by aetiology and fibrosis stage, so the same value carries different meaning in hepatitis B cirrhosis (35), in hepatitis C cirrhosis after sustained virological response (8), and in MA SLD (31). The viral and fibrosis markers reviewed in Section 4 are precisely the variables that define this context. Aetiology-spanning marker sets (13) and aetiology-stratified models are needed, with MA SLD-associated HCC treated as a distinct context given its non-cirrhotic, AFP-poor presentation (18, 31).

### 5.5 Integration — a personalised longitudinal risk model

The three axes can be unified in a per-patient model that continuously updates each individual’s aetiology-conditioned tumour–host equilibrium from surveillance data and flags malignant emergence as a multidimensional anomaly (**Figure 3B**). We deliberately name this construct a personalised longitudinal risk model. It is a continuously updated statistical representation of an individual’s longitudinal biomarker state and its expected stable range — in effect a personalised reference interval that learns over time — and not a mechanistic, physics-based simulation of hepatic physiology. New measurements are scored for anomaly against the patient’s personal baseline and its permissible fluctuation. The construct requires no biophysical model of the liver to be useful; it is closer to the per-patient prediction models and nomograms already familiar in HCC (48), and **Figure 3B** should be read in that sense. The machine-learning and multi-omics methods the field is already adopting (18, 27, 28) are well suited to this task — provided they are pointed at personalised anomaly detection on longitudinal trajectories rather than population classification on cross-sectional panels. The two clinical outputs are earlier detection (including small and AFP-negative tumours) and finer stratification — for risk, for aetiologic subtype, and for prediction of treatment response, where host-side markers are already informative (17, 21, 34).

### 5.6 A minimal formal description

The three axes can be stated compactly—not to prescribe a particular algorithm, but to make the framework precise and testable. Let each surveillance visit for patient i at time t yield a state vector xit=(sit,hit) of tumour-shed and host-response measurements, where sit collects tumour-shed measurements (for example, AFP, AFP-L3, DCP and cfDNA features) and hit host-response measurements (inflammatory indices, cytokines, autoantibody or repertoire read-outs). The fixed context ci (for example, aetiology and fibrosis stage) enters as a conditioning variable rather than as part of the state. A tumour-free reference distribution is estimated from a clinically matched cohort and the patient’s own stable longitudinal history, with partial pooling used to borrow information across similar patients. This yields a patient-specific baseline distribution ϕi=p(x∣tumour-free,ci), characterized by an estimated mean μ^​i​ and regularised covariance Σ^i. Thus, μ^i and Σ^i should not be interpreted as the simple sample mean and covariance of observations from patient i, but as partially pooled estimates that combine individual longitudinal information with the corresponding reference distribution learnt from clinically similar patients in the cohort. Malignant emergence is then scored not as a class-membership probability but as an anomaly—a departure of the current state from this baseline. One generic score is the Mahalanobis distance.


dit= √[(xit−μ^i)T∑^i-1(xit−μ^i)],

which reduces to the absolute within-patient z-score in one dimension and incorporates the learnt tumour-host covariance through the off-diagonal terms of Σ^i. A change along a direction that is common under the baseline covariance contributes less to the distance than a comparably sized change in an unusual direction, including potential departures from the usual joint tumour-host pattern. The Mahalanobis distance is therefore a generic multivariate anomaly score rather than a direct measure of tumour-host coupling; more specific measures could quantify changes in cross-compartment covariance or residual dependence between the axes. Divergence can accordingly be operationalised in several ways—as distance from baseline, a change-point in the multivariate trajectory, a change in covariance or dependence structure between the axes, or the residual from a fitted dynamical model—and the optimal implementation is an empirical question. The temporal axis enters as repeated observations progressively inform the partially pooled baseline and the resulting anomaly trajectory; the contextual axis enters through conditioning on ci; and the compartmental axis enters through the joint modelling of tumour-shed and host-response measurements. In this notation, the framework’s central prediction (Section 7.1) is explicit: a score based on the full joint state xit and its longitudinal history should detect early malignancy better than one based on the tumour-shed sub-vector sit alone, after accounting for ci and relevant clinical covariates.

## 6 Challenges and limitations

Realising the framework will require longitudinal biobanking, harmonised repeated sampling on a stable analytical platform, enough serial measurements to learn a reliable personal baseline, and careful control of lead-time and length-time biases; the principal clinical obstacle is the non-specificity of host-response signals, which must be modelled as explicit covariates rather than ignored. Our landscape analysis is itself based on titles and abstracts and is therefore indicative of relative research effort rather than exact, although the central imbalances are large and robust to plausible misclassification. Extended limitations, the confounder-handling strategy and the corpus-analysis caveats are set out in the **Supplementary Material**.

## 7 Discussion

Blood-biomarker research in HCC has reached a plateau not for want of molecules but because of how those molecules are measured and interpreted: once, from one compartment, against one population threshold, regardless of aetiology. Reframing early detection as personalised anomaly detection, and organising biomarkers along temporal, compartmental and contextual axes, offers a way past that plateau using assays that largely already exist. The approach is deliberately aimed at the patients who are currently failed — those with early-stage and AFP-negative tumours — and is directly testable in the surveillance cohorts that already generate the longitudinal data it requires.

### 7.1 How the tumour–host divergence hypothesis can be tested

The framework is deliberately falsifiable, and its claims reduce to four predictions that can be evaluated against the current best-in-class composite, GALAD, in matched designs. (P1) A paired, coupling-aware model that reads the relationship between a tumour-shed axis and a host-response axis adds incremental early-stage discrimination over a well-constructed single-compartment longitudinal model, such as a longitudinal application of GALAD; if it adds nothing, the central claim is wrong. (P2) For at least a subset of tumours, a sustained departure of the host-response axis from the patient’s own baseline precedes, or coincides with, the point at which tumour-shed markers cross a conventional threshold, widening the detectable early window. (P3) Scoring each patient against their personal longitudinal baseline detects small and AFP-negative tumours that a fixed population threshold misses. (P4) Conditioning the baseline on aetiology and fibrosis stage — metabolic dysfunction-associated steatotic liver disease in particular — improves discrimination over aetiology-blind interpretation. Each prediction names its comparator, its population (early-stage and AFP-negative disease) and the direction of the expected effect, and each can be tested first retrospectively, in surveillance cohorts with banked serial samples, and then prospectively. The strongest early test is also the least expensive: pairing an existing tumour-shed panel with a single host-response read-out — an autoantibody, immune-repertoire or inflammatory-index measure — in a cohort already collecting longitudinal samples, and asking whether the paired, trajectory-based reading outperforms the tumour-shed panel alone.

### 7.2 When the hypothesis may fail

Stating how the framework could be refuted matters as much as stating what it predicts. Three failure modes would falsify or substantially weaken it. First, if the added host-response and temporal features behave merely as further surrogates of tumour burden — sharing the failure mode of GALAD’s oncofetal and coagulation inputs — they supply no incremental conditional information and yield no gain; the framework’s value rests on the added signals retaining incremental predictive information after conditioning on tumour burden, aetiology, fibrosis stage and relevant clinical covariates. Second, host-response signals are non-specific: intercurrent infection, biliary or hepatitic flares, decompensation, vaccination, changes in alcohol intake and shifting metabolic comorbidity all perturb the host axis. The framework’s defence is that coupling (an isolated inflammatory insult moves only the host axis, whereas malignancy is predicted to produce a sustained departure of the joint tumour–host trajectory from its usual coupling pattern) and temporality (a self-limiting event returns to baseline, whereas a tumour produces a sustained divergence) will separate these from incipient cancer; whether these safeguards hold specificity at a clinically acceptable level against the frequent intercurrent events of cirrhosis is itself an empirical question, and if they do not, the false-positive rate will be prohibitive. Third, the approach presupposes that enough serial measurements can be obtained, on a stable analytical platform, to learn a reliable personal baseline; where within-person variability is large or sampling is sparse, no stable baseline can be defined and the anomaly signal disappears. Any claim of earlier detection must also survive correction for lead-time and length-time bias before it can be believed.

### 7.3 A pathway to clinical implementation

If the early tests are supportive, translation should follow the phased logic of established biomarker development rather than a single leap to clinical use. The necessary substrate — longitudinal biobanks with harmonised, repeated sampling — already exists in several surveillance cohorts, which makes a retrospective, nested proof of concept feasible now: reconstruct per-patient trajectories, fit an aetiology-conditioned tumour–host model, and test the four predictions against banked outcomes. Analytically, the machine-learning and multi-omics methods the field is already adopting are well suited to the task, provided they are directed at personalised anomaly detection on longitudinal trajectories rather than population classification on cross-sectional panels; the proposed personalised longitudinal risk model is no more than a continuously updated personal reference interval, not a mechanistic simulation of the liver. Prospective validation, ideally embedded in ongoing screening trials, would then need to demonstrate not only discrimination but a stage shift towards curable disease, while addressing generalisability across aetiologies and populations, interpretability for clinicians, cost and regulatory acceptance. None of these steps requires a new analyte; each requires only that existing measurements be paired, tracked and interpreted against the individual — the practical claim at the centre of the hypothesis.

Clinical feasibility will depend heavily on the quality of the baseline estimate. Several serial samples obtained on a harmonised analytical platform will be needed, while real surveillance data will inevitably include missing visits and unequal sampling intervals. The model must therefore accommodate incomplete and irregular series. It must also distinguish gradual changes related to fibrosis progression, ageing, medication, metabolic disease or liver function from changes associated with malignant emergence. This distinction may be possible when background drift is slow and the tumour-related change is sustained, but it will become unreliable when the available history is short or intrinsically variable. Mixed-effects, state-space, Bayesian change-point and Gaussian-process models all provide possible approaches to this problem. Their relative performance in HCC surveillance, including the number of observations needed before a useful personal baseline can be defined, will have to be established empirically.

## Data Availability

The original contributions presented in the study are included in the article/**Supplementary Material**. Further inquiries can be directed to the corresponding author.

## Supplementary material

Supplementary Data Sheet The de-duplicated corpus, the per-record classification, the 23-class taxonomy dictionary, the 200-record validation sample, and the keyword-scoring script.
