# Supplementary material for: Tumour–host divergence: a hypothesis for personalised blood-based early detection of hepatocellular carcinoma
Source: Front Oncol. 2026 Sep 17;16:1932525. doi: 10.3389/fonc.2026.1932525 (PMC13627005; doi:10.3389/fonc.2026.1932525)
Supplement: Supplementary file 1 [file Supplementaryfile1.docx]

# Supplementary file 1

# Supplementary Note S1. Extended limitations and corpus-analysis caveats

Realising the framework requires longitudinal biobanking and harmonised, repeated sampling on a stable analytical platform; enough serial measurements to learn a reliable personal baseline; and careful control of lead-time and length-time biases when claiming earlier detection. The single largest clinical obstacle is the non-specificity of host-response signals. Intercurrent infection, biliary or hepatitic flares, decompensation, vaccination, changes in alcohol intake, drug exposure and shifting metabolic comorbidity all perturb the host axis and, if read cross-sectionally and in isolation, would inflate the false-positive rate. The framework does not assume these confounders away; it addresses them by design in three ways. First, the temporal axis distinguishes a transient, self-resolving excursion from a sustained, progressive divergence, so a single inflamed time point does not trigger an alarm. Second, the compartmental axis requires correlated movement of both axes, which an isolated inflammatory event does not produce. Third, intercurrent events that are documented — infection, flare, anticoagulation, abstinence, weight change — should enter the per-patient model as explicit covariates rather than be left as unmodelled noise. Whether these safeguards are sufficient to hold specificity at a clinically acceptable level is itself one of the empirical questions the proposed studies are designed to answer, and we present it as a hypothesis to be tested, not a solved problem. Personalised, multi-compartment models additionally raise issues of generalisability, interpretability, cost and regulatory acceptance, and must be validated prospectively before clinical use.

Our landscape analysis has its own limitations, which we state plainly. It draws on titles and abstracts rather than full texts and therefore yields counts that are indicative of relative research effort rather than exact; full-text and embedding-based classification would refine the proportions, and our deliberately granular twenty-three-class taxonomy gives figures that differ from broader earlier groupings. To make the analysis reproducible and auditable we provide the exact search strings, database export dates, the de-duplication rule and the complete keyword dictionary in the Supplementary Data. To quantify the reliability of the keyword taxonomy rather than merely assert it, a random sample of 200 records was classified independently by two reviewers reading the full texts; disagreements between the two reviewers were resolved by consensus to form the reference standard against which the automated keyword assignment is scored. The keyword taxonomy agreed with this adjudicated full-text reference at Cohen’s κ = 0.81; per-compartment sensitivity and specificity are reported below (Supplementary Table 1) for the tumour-shed, host-response, bridge and methodological compartments. Notably, host-response classification showed high specificity but only moderate sensitivity (69.4%), confirming that a title/abstract screen tends to under-capture host-response work and that the tumour-versus-host imbalance is therefore conservative. The labelled sample together with the scoring script are released as Supplementary Data so that these figures are fully reproducible and the taxonomy can be independently re-audited. As noted in Section 1, the residual error is asymmetric: host-response work is the most likely to be missed by a title/abstract screen, so the dominant tumour-versus-host imbalance is conservative. These refinements are in any case unlikely to overturn the central imbalances — tumour-centricity, the prognosis-over-detection skew, and the near-absence of longitudinal and paired designs — which are large and robust to plausible misclassification. Finally, the framework reorganises and recombines existing markers; it does not assert that any specific marker pair is optimal — that, too, is an empirical question the proposed studies are designed to answer.

**Supplementary Figure 1. De-duplication workflow for the biomarker landscape analysis**. PubMed (6,711 records) and Embase (2,236 records) exports for the query “hepatocellular carcinoma blood diagnostic biomarker” were pooled (8,947 raw records); 878 duplicates were removed (records sharing a DOI, or lacking a DOI but sharing a normalised title), leaving 8,069 unique records, which were then classified into the 23-class taxonomy shown in Figure 1A. Counts in Figure 1A and throughout this manuscript derive from this de-duplicated corpus.

| **Compartment** | **Sensitivity vs full text** | **Specificity vs full text** |
| --- | --- | --- |
| Tumour-shed | 98.0% (96/98) | 81.4% (83/102) |
| Host-response | 69.4% (25/36) | 98.8% (162/164) |
| Bridge / dual-compartment | 100% (2/2) | 100% (198/198) |
| Methodological | 75.0% (3/4) | 100% (196/196) |

**Supplementary Table 1. Reliability of the keyword taxonomy against a full-text reference standard, assessed on a random sample of 200 records (164 PubMed, 36 Embase).** Two reviewers independently classified each record from its full text; disagreements were resolved by consensus to form the reference standard. Agreement between the keyword taxonomy and this adjudicated full-text reference was Cohen’s κ = 0.81 across the five-category scheme. Per-compartment sensitivity and specificity against the full-text reference are entered above, with the numerator and the reference-standard denominator shown in parentheses. The bridge/dual-compartment and methodological estimates rest on very few reference-positive records (n = 2 and n = 4, respectively) and are therefore imprecise; the host-response sensitivity of 69.4% confirms that a title/abstract screen under-captures host-response work, consistent with the conservative direction of the tumour-versus-host imbalance noted in Sections 1 and 6. The labelled sample and the scoring script are provided as Supplementary Data to allow independent re-audit.
